# Vascular Calcification: New Insights Into BMP Type I Receptor A

**DOI:** 10.3389/fphar.2022.887253

**Published:** 2022-04-06

**Authors:** Zhixing Niu, Guanyue Su, Tiantian Li, Hongchi Yu, Yang Shen, Demao Zhang, Xiaoheng Liu

**Affiliations:** ^1^ State Key Laboratory of Oral Diseases, West China Hospital of Stomatology, Sichuan University, Chengdu, China; ^2^ West China School of Basic Medical Sciences & Forensic Medicine, Sichuan University, Chengdu, China

**Keywords:** vascular calcification, BMPR1A, VSMCs, ECS, atherosclerosis

## Abstract

Vascular calcification (VC) is a complex ectopic calcification process and an important indicator of increased risk for diabetes, atherosclerosis, chronic kidney disease, and other diseases. Therefore, clarifying the pathogenesis of VC is of great clinical significance. Numerous studies have shown that the onset and progression of VC are similar to bone formation. Members of the bone morphogenetic protein (BMP) family of proteins are considered key molecules in the progression of vascular calcification. BMP type I receptor A (BMPR1A) is a key receptor of BMP factors acting on the cell membrane, is widely expressed in various tissues and cells, and is an important “portal” for BMP to enter cells and exert their biological effect. In recent years, many discoveries have been made regarding the occurrence and treatment of ectopic ossification-related diseases involving BMP signaling targets. Studies have confirmed that BMPR1A is involved in osteogenic differentiation and that its high expression in vascular endothelial cells and smooth muscle cells can lead to vascular calcification. This article reviews the role of BMPR1A in vascular calcification and the possible underlying molecular mechanisms to provide clues for the clinical treatment of such diseases.

## Introduction

Vascular calcification (VC) is an important indicator of increased risk for diabetes, atherosclerosis, chronic kidney disease (CKD), and other diseases. It is also a key factor for the high morbidity and mortality of cardiovascular and cerebrovascular diseases (Demer and Tintut, 2008; Schenker et al., 2008; Shroff and Shanahan, 2010). VC is characterized by the deposition of mineral calcium as a calcium-phosphate complex in the vascular wall system; its pathogenesis and progression are very similar to those of bone formation (Lee et al., 2020). Both processes involve the activation of bone matrix proteins, calcification-inducing factors, and various signaling pathways in diverse cell types, including vascular smooth muscle cells (VSMCs), macrophages, and endothelial cells (Ecs). At present, our understanding of the pathogenesis of VC includes aging, chronic inflammation, calcium-phosphorus imbalance, oxidative stress, and mitochondrial dysfunction. Recently, the bone-vascular axis theory has provided a new paradigm for the study of VC. It has been found that the ectopic calcification of the vessel wall is often associated with decreased bone density or disturbances in bone metabolism. This phenomenon occurs mostly in postmenopausal women and patients with chronic kidney disease and osteoporosis (Persy and D’haese, 2009; Gu et al., 2020). Based on the theory of bone-vascular axis, many studies have reported the role of BMP ligands in vascular calcification, while BMPR1A, the key receptor of BMPs, has rarely been reported. Studies have confirmed that BMPR1A is necessary for chondrogenesis and osteogenesis, and its overexpression in vascular smooth muscle cells can lead to VC (Mang et al., 2020; Yang et al., 2020). Although BMPR1A has been proposed to be involved in vascular calcification, the specific mechanism remains unclear. In this review, we focus on the role of BMPR1A in vascular calcification and the possible underlying molecular mechanisms to explore its potential therapeutic implications. First, we discussed VC and BMPR1A signaling, followed by its effects on ECs and smooth muscle cells (SMCs), and how an imbalance in BMPR1A signaling leads to VC.

## Vascular Calcification

### Inducing Factors and Mechanism of Vascular Calcification

**FIGURE 1:**
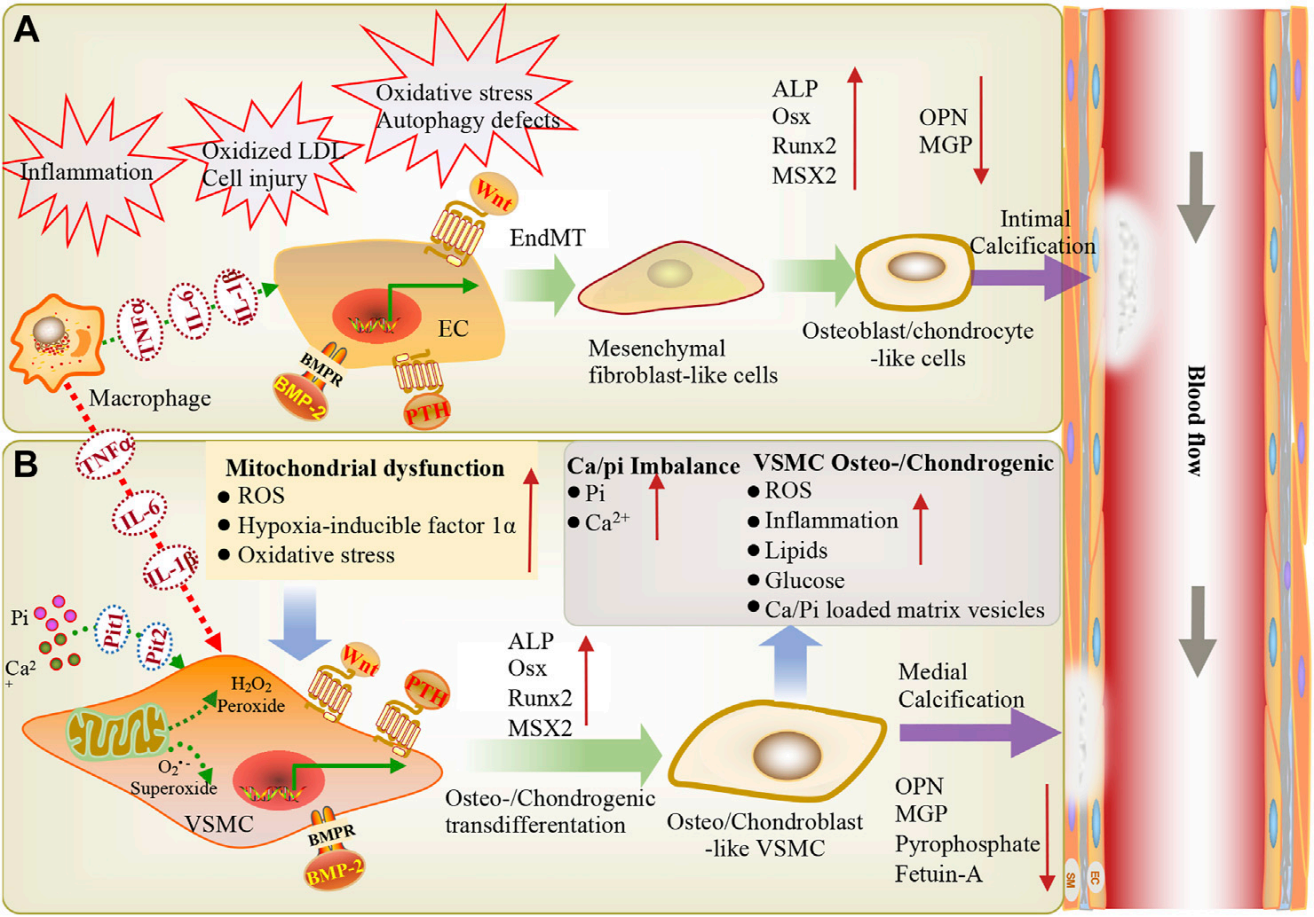
During inflammation, macrophages infiltrate through EC adhesion and migration across endothelial cells, resulting in macrophage activation and the release of inflammatory cytokines (TNF-α, IL-6, IL-1β), further stimulating VSMCs and ECs, eventually leading to vascular calcification. Vascular endothelial cells exposed to shear stress induced by blood flow are more sensitive to shear stress-induced EndMT and BMP-induced osteogenic differentiation, thereby triggering endothelial damage and inducing vascular calcification. **(A)** Endothelial cells can be stimulated via inflammation, oxidative LDL aggregation, cell injury, oxidative stress, and autophagy defects. BMP, PTH and Wnt signaling are involved in endothelial mesenchymal transformation, the differentiation of endothelial cells into mesenchymal fibroblasts, and their further differentiation into osteoblast-like cells. These changes are accompanied by a high expression of osteogenic genes (ALP, Runx2, BMP2, MSX2) and low expression of the calcification inhibitor MGP, leading to intimal calcification. **(B)** Ca^2+^/P_i_ imbalance and mitochondrial dysfunction lead to reactive oxygen species production and oxidative stress. BMP, Wnt, and PTH signaling induce the osteogenic/chondrogenic differentiation of vascular smooth muscle cells, ultimately leading to medial calcification. EC: endothelial cells; VSMC: vascular smooth muscle cell; ALP: alkaline phosphatase; Pit1, Pit2: NaPi cotransporters; LDL: low-density lipoprotein; OPN: osteopontin; PTH: parathyroid hormone; Runx2: runt-related transcription factor-2; Msx2: msh homeobox-2; IL1-β: interleukin-1 beta; IL-6: interleukin-6; Osx: osterix; EndMT: endothelial-mesenchymal transition; BMP: Bone morphogenetic protein; ROS: Reactive oxygen species; TNF-α: tumor necrosis alpha; Pi: inorganic phosphate.

VC is a complex process, and numerous *ex vivo* and *in vivo* studies have confirmed that VC is very similar to bone formation in terms of pathogenesis and progression (Figure 1) (Neven et al., 2011; Andrews et al., 2018; Durham et al., 2018). In the past few decades, the inducing factors of vascular calcification have been continuously proposed (Table 1). The interference of internal and external environments, such as aging, inflammation, diabetes, chronic kidney disease, oxidative stress, and mitochondrial dysfunction, can lead to VC. It has been reported that inflammation-related tumor necrosis factor (TNF-α) and interleukin-1 beta (IL-1β) can induce endothelial-mesenchymal transition (EndMT) by regulating BMPRs. These then induce osteogenic differentiation through the BMPR-JNK signaling axis, promoting vascular calcification (Sanchez-Duffhues et al., 2019). In diabetes, hyperglycemia and an imbalance in mineral ion homeostasis could lead to endothelial cell injury. Calcified vascular cells (CVCs) perceive extracellular damage signaling and induce CVCs to differentiate into osteoblast-like cells by upregulating osteogenic factors and activating Wnt signaling, promoting vascular calcification (Bartoli-Leonard et al., 2018). In chronic kidney disease, calcium and phosphorus imbalance can lead to mitochondrial dysfunction, increase the release of reactive oxygen species, trigger oxidative stress and inflammatory responses, and induce the reverse differentiation of VSMCs into osteoblast-like cells, leading to vascular calcification (Zhu et al., 2020; Phadwal et al., 2021). At the cellular level, when the structure and function of vascular endothelial cells are abnormal, the expression of proinflammatory cytokines TNF-α and IL-1β can induce EndMT *via* BMP signaling, thereby inducing osteogenic differentiation and promoting vascular calcification (Sanchez-Duffhues et al., 2019). After being subjected to biological stress or injury, VSMCs can regulate the level of contractile proteins and reconstruct the extracellular matrix (ECM) or differentiate into osteoblast/chondrocyte-like cells to induce vascular calcification (Durham et al., 2018; Pescatore et al., 2019; Lu et al., 2020).

**TABLE 1:** Inducing factors and the mechanism of vascular calcification.

Inducing factors	Mechanism	References
Calciprotein particles	Phosphate/calcium homeostasis disorders and changes in hormone levels (high FGF23, low vitamin D activity, and high parathyroid hormone)	Kuro, (2021)
Oxyphospholipids and their mediators	Oxyphospholipids in macrophages promote the assembly of inflammatory bodies and the production of cytokines with calcification-promoting properties. Macrophages promote and/or enhance plaque mineralization by generating extracellular vesicles	Chignon et al. (2021)
Platelet	Platelets involved in thrombosis release various bioactive molecules, some of which have calcification-promoting properties. Signal crosstalk between platelets and vascular/valve cells can promote ectopic mineralization	Schurgers et al. (2018)
Aging	Aging causes mitochondrial dysfunction and increased ROS production, activates inflammation, increases oxidative stress, upregulates BMPs and enhances the expression of the osteogenic transcription factor Runx2, which in turn promotes vascular calcification	Kwon et al. (2017)
Inflammation	By releasing proinflammatory cytokines, endothelial cells are induced to transform into mesenchymal cells, and vascular smooth muscle cells are reversely differentiated into osteoblasts, thereby promoting vascular calcification	Sanchez-Duffhues et al. (2019)
Diabetes	Hyperglycemia and an imbalance in mineral ion homeostasis lead to endothelial cell injury. The medial mucosal layer responds by triggering the repair response. CVCs perceive extracellular signals, upregulate osteogenic factors, downregulate sirtuin-1, and activate Wnt signaling, resulting in CVC differentiation into osteogenic cells and promoting vascular calcification	Bartoli-Leonard et al. (2018)
CKD	A decrease in fetal globulin A and pyrophosphate levels, increase in serum phosphate levels (hyperphosphatemia), hyperparathyroidism, and PTH and FGF23 deficiency could lead to calcium and phosphate imbalance and promote vascular calcification	Chen et al. (2020)
Hypertension	Blood pressure fluctuation changes the production of ATP, increases ROS, and disturbs the mitochondrial network in VSMCs, leading to mitochondrial dysfunction and eventually VC.	Bartolak-Suki and Suki, (2020)
Dyslipidemia	Polarization of induced proinflammatory (M1) function in the monocyte/macrophage system leads to an increased release of proinflammatory cytokines (e.g., IL-6, IL-1β, and TNF-α) and the production of reactive oxygen species, which in turn induce the calcification of VSMCs and ECs	Torres-Castro et al. (2016)
High phosphate	High phosphate levels can directly promote VSMC calcification, leading to VSMCs transforming from the contractile to osteochondral phenotype	Bai et al. (2021)
Klotho deficiency	Klotho deficiency leads to the upregulation of BMP2, BMP4, and Runx2 expression and promotes BMP2/VitD3-induced osteogenic transdifferentiation of VSMCs, leading to vascular calcification	Lin and Sun, (2021)

The BMP signaling pathway is vital for osteogenesis, EndMT, and VSMC calcification. Several treatment strategies for vascular calcification related to the BMP signaling pathway have been proposed. For example, BMP antagonists (LDN-193189, which targets the BMP type I receptor; or BMPRIA-Fc, which targets the BMP ligands) inhibit the BMP signaling pathway, which can reduce vascular calcification and improve the survival rate of matrix Gla protein-knockout mice (Malhotra et al., 2015). Furthermore, overexpression of the *MGP* gene inhibits the BMP signaling pathway and reduces BMPR I receptor expression, thereby reducing the formation of vascular calcification in apolipoprotein E-knockout (ApoE^−/−^) mice (Nakagawa et al., 2010; Yao et al., 2010; Ducy et al., 2015; Malhotra et al., 2015). The inhibition of BMP signaling by a high-fat diet reduces intimal calcification in low-density lipoprotein receptor (LDLR)-deficient mice (Derwall et al., 2012). Dorsmorphin homologous 1 inhibits the phosphate-induced differentiation of VSMCs to osteoblast-like cells by inhibiting BMP-2 (Lin et al., 2017). Gla Rich Protein (GRP) interacts with BMP-2 and the BMP-SMAD signaling pathway, inhibiting the BMP-2 signaling pathway and SMAD1/5/8 phosphorylation *via* noggin and dorsmorphin, respectively, to reduce VSMC calcification and bone/cartilage gene expression (Willems et al., 2018).

### Intimal and Medial Calcification

Depending on the mechanism of formation and location, VC can be classified as intimal or medial (Yang et al., 2020). Intimal calcification occurs in the inner layer of the blood vessel wall, in a process very similar to endochondral osteogenesis. It mainly occurs during atherosclerosis, which is caused by lipid accumulation, macrophage invasion, smooth muscle cell migration, and the proliferation or transdifferentiation of osteoblast-like cells. Medial calcification (also known as “Monckeberg’s medial sclerosis”) occurs in the medial layer, and it is commonly observed in aging patients or those with diabetes, CKD, or dyslipidemia. It is also related to mineral metabolism disorders, similar to intramembranous bone formation (Villa-Bellosta and O'Neill, 2018; Chen et al., 2020). In general, the process of these two types of calcification is similar to the transformation of smooth muscle cells into bone and cartilage-like cells through calcium deposition in VSMCs (Lee et al., 2020). Reduced vascular compliance is a common characteristic of both types of calcification. Whether plaque is formed *via* atherosclerosis, calcification, or medial mineralized crystal deposition, the common molecular signaling pathways for both processes include the regulatory protein BMP2 and the transcription factor Runx2, which drive the bone formation process (Thompson and Towler, 2012; Lee et al., 2020).

## The Role of BMP in Vascular Calcification

### BMP Signaling

BMP signaling is essential for the development of organisms and the stability of the internal environment. It was initially identified by its ability to induce bone and cartilage formation but was later found to be highly expressed during vascular calcification (Cai et al., 2012; Morrell et al., 2016). BMPs are members of the transforming growth factor beta (TGF-β) family of proteins and are expressed in various cells. They bind to receptors *via* ligands to form receptor complexes composed of type I receptors (ALK2, ALK3, and ALK6) and type II receptors (BMPR2, ACVR2A, and ACVR2B) and are widely involved in signal transduction and molecular regulatory processes. BMPs are usually produced by endothelial cells, osteoblasts, and chondrocytes in the bone. However, some studies have found that there are some BMPs in serum samples, indicating that they may circulate in the blood to act at the systemic level (Cheng et al., 2004; Simic et al., 2006). BMP signaling cannot be separated from the interaction between ligands and receptors. Upon the binding of BMP ligands to their respective receptors, type II receptors phosphorylate and activate type I receptors, which in turn phosphorylate intracellular signaling molecules, activating SMAD-dependent and SMAD-independent signaling pathways. The canonical BMP-SMAD-dependent signaling pathway activates R-SMADs (R-SMAD1, R-SMAD5, R-SMAD 8) *via* type I and type II receptors, which then bind to SMAD4 to form a complex that translocates to the nucleus, where it binds to DNA and interacts with transcription factors to regulate transcription of downstream target genes (Miyazono et al., 2005; Sieber et al., 2009; Luo et al., 2010; Cai et al., 2012). BMP-SMAD-independent signaling pathways include the ERK, JNK, and p38 MAPK signaling pathways, whose main roles include regulating apoptosis, epithelial-mesenchymal transition, cell migration, cell proliferation and differentiation, and the extracellular matrix (Yu, et al., 2002). In addition, type I BMP receptors are widely expressed in various cell types, and the affinity of BMPs for type I receptors is higher than that for type II receptors (Lavery et al., 2008). BMP2 was initially found to be an inducer of ectopic bone and can be detected in both the cartilage and bone (Urist, 1965; Nomura and Yamamoto, 2000). BMP2 can stimulate bone formation through the BMP receptor, which preferentially interacts with BMPR1A compared to other receptors (Kai et al., 2009; Mang et al., 2020).

### BMPR1A in BMP Signaling

BMPR1A encodes type I TGF-β family receptors in the BMP2 and BMP4 signaling pathways (Mishina et al., 1995). Likewise, BMPR1A is also a serine/threonine kinase transmembrane protein type I receptor expressed in many tissues and plays a critical role in angiogenesis and the regulation of vascular homeostasis (Shi and Massagué, 2003; Lee et al., 2017; Sanchez-de-Diego et al., 2019). BMPR1A is the main type I receptor that transduces BMP signaling in preosteoblasts and has a higher ability to transduce BMP-SMAD signaling than BMPR1B or ACVR1 (Pan et al., 2017). In the absence of BMPR1A, the phosphorylation of Smad decreases while the activation of p38 and Erk increases, changing the balance of the BMP signaling cascade. Studies have shown that BMPR1A deficiency can lead to premature osteoblast differentiation and intramembranous ossification, which is necessary for cartilage formation and osteogenesis (Shi et al., 2017; Mang et al., 2020). Additionally, the inactivation of BMPR1A can lead to the excessive activation of downstream signaling by other BMPI receptors (T. Maruyama et al., 2021). According to a previous study, a lack of BMPR1A will impair the self-renewal, clonal expansion, and osteogenic ability of suture stem cells and plays an important role in inhibiting cell differentiation or promoting asymmetric cell division (T. Maruyama et al., 2021). Another study indicated that increased BMPR1A expression enhances the adipogenic differentiation of mesenchymal stem cells, activating the BMP-pSmad1/5/8 signaling pathway, and contributing to abnormal fat metaplasia and new bone formation in patients with ankylosing spondylitis (Liu et al., 2019). Similarly, BMPR1A is also essential for BMP-induced phosphorylation of Smad1/5/8 in heart valve progenitors (Lockhart et al., 2014). During aortic valve calcification, deletion of BMPR1A or inhibition of its activity can lead to absence of the BMP signaling pathway, resulting in the loss of osteochondrogenic features and blocked calcified nodule formation, thereby preventing aortic valve calcification (Gomez-Stallons et al., 2016). Thus, BMPR1A signaling plays an essential role in restricting preosteoblast proliferation and promoting osteoblast activity.

### Role of BMP Signaling in the Bone-Vascular Axis

The bone is not only a site of mineral storage but also the main site for stem cell maintenance and hematopoiesis. It plays a dual role in maintaining the vascular integrity of endothelial progenitor cells and aggravating VC *via* inflammatory cells (Kim et al., 2020). Numerous studies have shown that vascular calcification is highly similar to bone formation. The minerals deposited in the vascular wall are mainly alkaline calcium phosphate in the form of apatite, including hydroxyapatite [Ca_10_ (PO_4_)_6_ (OH) _2_ ] crystals, consistent with the composition of bone (Reynolds et al., 2004). Some secreted proteins that connect the bone and blood vessels play important roles in vascular calcification, such as receptor activator for nuclear factor kB (RANK)/RANK ligand (RANKL)/osteoprotegerin (OPG), BMPs, Runx2, osterix (OSX), fetuin-A, and MGP (Pereira and Frazao, 2020). Some studies have also shown that there is an independent negative correlation between bone mineral density and atherosclerosis and that patients with osteoporosis are more prone to aortic and carotid calcification (Hofbauer et al., 2007; Anagnostis et al., 2009; Zhang et al., 2016). In addition, disorders of bone volume and bone turnover in patients with uremia may increase the risk of VC and eventually lead to an extremely high risk of cardiovascular death (Adragao et al., 2009). Some clinical experiments have confirmed that bisphosphonates can inhibit vascular calcification while preventing fracture risk during the treatment of osteoporosis (Kranenburg et al., 2016). Recent studies have shown that BMP-induced miRNAs can regulate the proliferation and migration of VSMCs and induce their phenotypic conversion and that their abnormal regulation can lead to various vascular diseases (Park and Kang, 2020). Therefore, this evidence suggests that crosstalk exists between the bone and the vascular system.

## Role of BMPR1A in Vascular Calcification

### Role of BMPR1A in the Osteogenic Differentiation of Endothelial Cells

ECs are an essential component of blood vessels and one of the key factors for maintaining normal cardiovascular function. They are highly active cell monolayers that can rapidly adapt to and respond to endogenous and exogenous signaling. Endothelial dysfunction has been observed in many cardiovascular diseases, including atherosclerosis (Widlansky et al., 2003). Currently, many researchers have studied VC as an outcome of atherosclerosis (Bjorklund et al., 2020). Inflammatory factors (such as TNF-α and IL-1β) and TGF-β family ligands (including BMPs) are jointly involved in the occurrence and development of calcified aortic plaques (Dhore et al., 2001; Gistera and Hansson, 2017). Furthermore, some studies have suggested that ECs can function as a special source of osteogenic progenitors during vascular calcification (Chen et al., 2015; Sanchez-Duffhues et al., 2015; Evrard et al., 2016).

**FIGURE 2:**
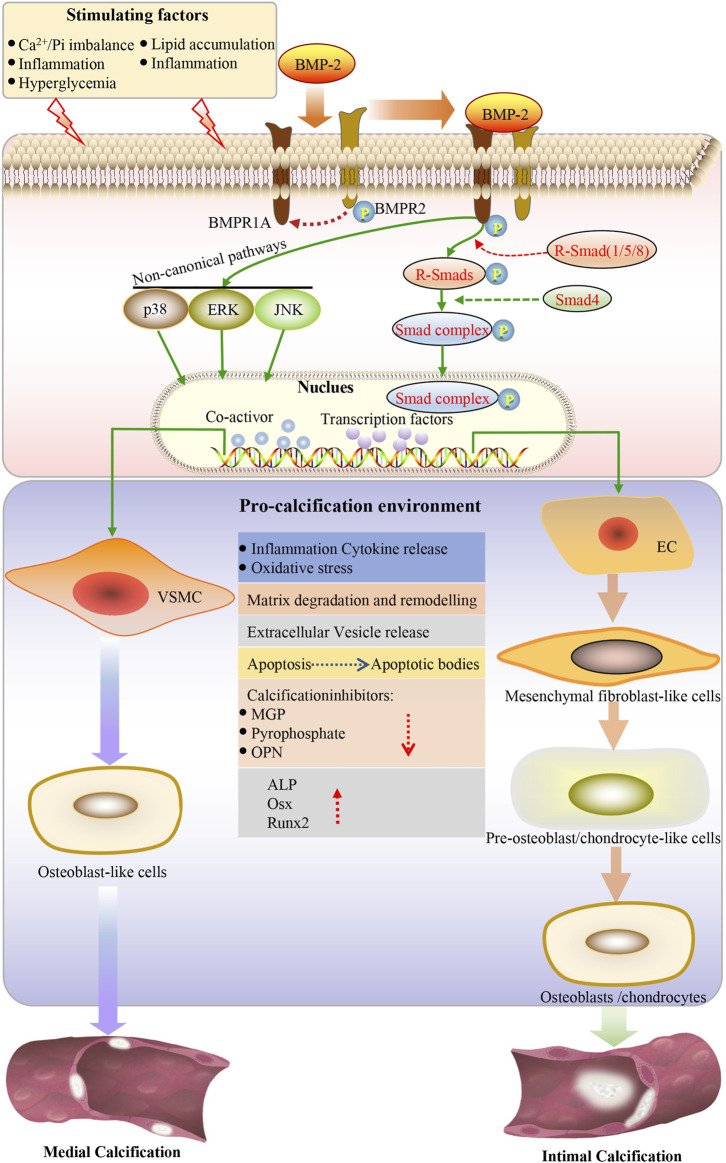
Under the action of stimulating factors, BMP2 binds to the receptor, the type I receptor is phosphorylated by the type II receptor, subsequently activating the classical BMP-SMAD and BMP-noncannonical pathway. SMAD1/5/8 is the effector of the BMP-SMAD pathway. Phosphorylated SMAD1/5/8 forms a heterogeneous complex with SMAD4 and is translocated to the nucleus, where it can regulate the expression of target genes by interacting with other transcription factors. Endothelial cells undergo endothelial mesenchymal transformation and differentiate into mesenchymal fibroblasts, which further differentiate into pre-osteoblasts and osteoblasts in a pro-calcified environment, leading to intimal calcification. VSMCs can be transformed into the bone/cartilage phenotype. Osteogenic/chondroblast-like cells actively promote calcification by reducing the expression of calcification inhibitors, increasing apoptosis, releasing apoptotic bodies and calcified vesicles, remodeling the extracellular matrix, degrading elastin, and releasing proinflammatory cytokines, ultimately increasing oxidative stress. The osteogenic transcription factor induces the expression of Runx2 and osterix in VSMCs. Osterix is upregulated by Runx2 and becomes fully activated. This creates an environment to promote calcification so that vascular calcification can be achieved. EC: endothelial cells; VSMC: vascular smooth muscle cell; ALP: alkaline phosphatase; OPN: osteopontin; Runx2: runt-related transcription factor-2; Osx: osterix; ROS: reactive oxygen species; BMP: bone morphogenetic protein; BMPR1A: BMP type I receptor A; BMPR2: type II receptor; SMAD: homolog of the *drosophila* protein, mothers against decap-entaplegic (MAD) and the *Caenorhabditis elegans* protein SMA; ERK: extracellular signal-regulated kinase; JNK: c-jun N-terminal kinase.

Endothelial cells can undergo a process called EndMT under inflammation, characterized by the loss of endothelial characteristics and the acquisition of fibroblast-like phenotypes, which eventually re-differentiate into osteogenic potential cells under the guidance of various stimuli such as BMPs (Figure 1A) (Pavićević et al., 2016; Sanchez-Duffhues et al., 2019). The BMP-Smad signaling pathway plays an important role in EndMT (Figure 2). Studies have confirmed that most BMPs promote EndMT through the canonical Smad-dependent pathway and that IL-1β can activate the BMP signaling pathway by upregulating the expression of BMP2 (Shanmugam et al., 2015; Evrard et al., 2016; Hong et al., 2020; Yuan et al., 2021). Under conditions such as hyperglycemia, inflammation, and aging, endothelial cells are stimulated to release extracellular vesicles (EVs) or extracellular matrix protein particles (EMPs), which are enriched in Ca^2+^ and BMP2, and act as nucleation sites for calcification to induce mineralization (Yuan et al., 2021). Previous studies have shown that TNF-α can increase the expression of BMP2 and the release of EMPs in ECs, thus promoting osteogenesis and the calcification of VSMCs (Csiszar et al., 2005; Buendia et al., 2015). It is well known that the BMP2 signaling cascade is initiated by activating BMPR1A (ALK3), BMPR1B (ALK6), and BMPRII (Medici et al., 2010). BMPR1A is widely expressed in endothelial cells, and its overexpression is closely related to the early development of vascular calcification (Vinuesa et al., 2015; Wei et al., 2018). Hong et al. (2020) showed that BMPR1A and BMP type II receptors (BMPR-II, Act-RIIB) could activate IL-1β-induced EndMT, thereby promoting the expression of Runx2 and OSX. Huang et al. (2018) also demonstrated that IL-6 could promote the cell surface translocation of BMPR1A and enhance the BMP-2-induced osteogenic differentiation of mesenchymal cells by amplifying BMP-Smad signaling.

An increasing number of studies indicate that ECs play a significant role in vascular calcification *via* EndMT, the autocrine/paracrine pathway, EC-derived extracellular vesicles, angiogenesis, and mechanotransduction. These findings further confirmed that BMPR1A could induce osteogenic gene expression during vascular endothelial cell mesenchymal transformation and promote calcification plaque production, further promoting vascular calcification.

### Role of BMPR1A in the Osteogenic Differentiation of Vascular Smooth Muscle Cells

VSMCs have multiple phenotypes, including osteogenic, contractile, and synthetic, which can be changed from one phenotype to another under certain conditions (Bardeesi et al., 2017). Vascular calcification is an active, complex, and tightly regulated biological process that is mainly related to the transformation of the contractile phenotype of VSMCs to osteoblast-like cells (McCarty and DiNicolantonio, 2014; Evrard et al., 2015). This phenotypic transformation of VSMCs is characterized by the upregulation of osteogenic genes, such as BMP2, Runx2, OSX, ALP, and osteopontin (O’Neill, 2017). VSMCs constitute the majority of the vascular wall, maintaining vascular structure and regulating blood pressure (Waldo et al., 2005). Similar to osteoblasts, VSMCs are derived from mesenchymal precursor cells and express BMP2 (Sun et al., 2017). Apoptosis, the release of extracellular vesicles, the absence of calcification inhibitors (e.g., MGP), senescence-associated DNA damage, and osteo/chondrogenic differentiation are considered the main mechanisms of vascular smooth muscle cells undergoing vascular calcification (Neven et al., 2007; Liu et al., 2013; Schurgers et al., 2013).

VSMCs are the predominant cell type involved in vascular calcification, which can transdifferentiate into the chondrocyte, osteoblast, and osteocyte phenotypes in a calcified environment (Figure 1B) (Shroff et al., 2008; Zhu et al., 2011). The BMP-Smad signaling pathway plays an essential role in the transdifferentiation of VSMCs into osteoblasts and chondrocytes (Figure 2). Previous studies have reported that BMPR1A and BMPRII are highly expressed in VSMCs and mediate the response of SMCs to BMP2. BMPR1A and BMPRII are the dominant complexes mediating Smad1/5/8 phosphorylation in response to BMP2 (Upton et al., 2008; Goumans et al., 2018). Evidence suggests that the BMP2-Smad1/5/8 signaling pathway is involved in the osteogenic differentiation of VSMCs (Broege et al., 2013; Kee et al., 2014). When VSMCs receive stimulation signaling, phosphorylated Smad1/5/8 can accelerate translocation from the cytoplasm to the nucleus and initiate the osteogenic differentiation of VSMCs, which eventually leads to the increased expression of OSX, Runx2, and alkaline phosphatase (ALP) (Wang et al., 2018; Willems et al., 2018). One study showed that BMP2 accelerated the atherosclerotic intimal calcification in transgenic ApoE^−/−^ mice (Nakagawa et al., 2010). Another study indicated that BMP2 could affect SMC marker (SM22a and SM a-actin) loss and the expression of genes associated with the osteoblast phenotype, such as msh homeobox-2, ALP, and osteopontin (OPN) (Bardeesi et al., 2017). Furthermore, BMP2 plays a vital role in promoting VSMC apoptosis, Reactive oxygen species (ROS) production, and inflammatory responses (Agharazii et al., 2015; Voelkl et al., 2019). The inhibition of BMP2 expression in a CKD mouse model significantly reduced the osteogenic differentiation of VSMCs, thereby reducing BMP2-induced mineralization (Nguyen-Yamamoto et al., 2019). Sun et al. (2017) showed that endogenous BMP2 is involved in the osteogenic differentiation of VSMCs and subsequent calcification under IL-6 induction. BMPR1A, a high-affinity receptor of BMP2, also acts as a bridge to promote vascular calcification and plays a crucial role in the transdifferentiation of VSMCs into osteoblasts or chondrocyte-like cells.

## Conclusion and Outlook

Vascular calcification maintains many characteristics of bone formation, including the need to co-activate BMP signaling family members to recruit osteogenic or chondrogenic progenitor cells to promote the development of calcified cardiovascular diseases. Various BMP ligands, receptors, and regulators play environmental, tissue-specific, and time-dependent roles in the recruitment and activation of progenitor cells produced *via* vascular calcification during the development of *in situ* bone. In this review, we focused on discovering how BMPR1A regulates vascular calcification in the BMP-Smad signaling pathway and how damaged vascular endothelial cells and smooth muscle cells lead to VC mediated by BMP signaling. Collectively, BMPs can bind to BMP receptors on VSMCs and endothelial cells, thereby accelerating medial or intimal calcification. BMPR1A, a type I receptor of the BMP-SMAD signaling pathway, can specifically bind to BMP2 and play an important role in activating BMP2-mediated osteogenic differentiation and chondrogenesis.

Although inhibitors targeting BMPI receptors have been developed, the lack of selectivity between their receptors, which can lead to off-target effects and adverse reactions, remains a problem. Therefore, it is essential to develop more specific inhibitors against BMPR1A that may lead to vascular calcification. Using small molecule synthesis, BMPR1A inhibitors that inhibit BMP2-Smad signaling may effectively reduce vascular calcification. Moreover, manipulating BMPR1A expression may be a novel therapeutic strategy to ameliorate the abnormal osteogenic differentiation observed in Vascular calcification. It is worth noting that bone diseases should also be considered in the treatment of vascular calcification based on the bone-vascular calcification theory. In the future, it will be necessary to further investigate the potential mechanism of BMPR1A in Vascular calcification to strengthen the theoretical basis for VC treatment.
